# Powerful use of automated prioritization of candidate variants in genetic hearing loss with extreme etiologic heterogeneity

**DOI:** 10.1038/s41598-021-99007-3

**Published:** 2021-09-30

**Authors:** So Young Kim, Seungmin Lee, Go Hun Seo, Bong Jik Kim, Doo Yi Oh, Jin Hee Han, Moo Kyun Park, So min Lee, Bonggi Kim, Nayoung Yi, Namju Justin Kim, Doo Hyun Koh, Sohyun Hwang, Changwon Keum, Byung Yoon Choi

**Affiliations:** 1 Department of Otorhinolaryngology-Head and Neck Surgery, CHA Bundang Medical Center, CHA University, Seongnam, Republic of Korea; 2 Department of Otorhinolaryngology-Head and Neck Surgery, Seoul National University Bundang Hospital, Seongnam, Republic of Korea; 3 R&D Center, ENCell Co. Ltd, Seoul, Republic of Korea; 4 3billion, Inc., Seoul, Republic of Korea; 5 Department of Otolaryngology-Head and Neck Surgery, Chungnam National University Sejong Hospital, College of Medicine, Chungnam National University, Daejeon, Republic of Korea; 6 Department of Otorhinolaryngology, Seoul National University College of Medicine, Seoul, Republic of Korea; 7 Department of Biological Sciences, Vanderbilt University, Nashville, USA; 8 Department of Biomedical Science, The Graduate School, CHA University, Seongnam, Republic of Korea; 9 Department of Pathology, CHA University, CHA Bundang Medical Center, Seongnam, Republic of Korea

**Keywords:** Genetics, Diseases, Health care, Medical research

## Abstract

Variant prioritization of exome sequencing (ES) data for molecular diagnosis of sensorineural hearing loss (SNHL) with extreme etiologic heterogeneity poses a significant challenge. This study used an automated variant prioritization system (“EVIDENCE”) to analyze SNHL patient data and assess its diagnostic accuracy. We performed ES of 263 probands manifesting mild to moderate or higher degrees of SNHL. Candidate variants were classified according to the 2015 American College of Medical Genetics guidelines, and we compared the accuracy, call rates, and efficiency of variant prioritizations performed manually by humans or using EVIDENCE. In our in silico panel, 21 synthetic cases were successfully analyzed by EVIDENCE. In our cohort, the ES diagnostic yield for SNHL by manual analysis was 50.19% (132/263) and 50.95% (134/263) by EVIDENCE. EVIDENCE processed ES data 24-fold faster than humans, and the concordant call rate between humans and EVIDENCE was 97.72% (257/263). Additionally, EVIDENCE outperformed human accuracy, especially at discovering causative variants of rare syndromic deafness, whereas flexible interpretations that required predefined specific genotype–phenotype correlations were possible only by manual prioritization. The automated variant prioritization system remarkably facilitated the molecular diagnosis of hearing loss with high accuracy and efficiency, fostering the popularization of molecular genetic diagnosis of SNHL.

## Introduction

Hearing loss is among the most common sensory impairments, with a prevalence estimated at ~ 1.33/1000 neonates in developed countries^1^. Genetic causes contribute to > 50% of congenital sensorineural hearing loss (SNHL)^2,3^, and genetic diagnosis of SNHL has risen as a critical practice for predicting hearing-rehabilitation outcomes, as well as for genetic counseling^4,5^. Hearing loss exhibits unique characteristics that provide a favorable environment for molecular genetic diagnosis. Specifically, SNHL is mostly a monogenic disorder and follows Mendelian inheritance^3^, with autosomal recessive (AR) and autosomal dominant (AD) inheritance accounting for ~ 80% and ~ 15% cases of genetic hearing loss, respectively^3^. However, challenges exist in popularizing genetic diagnosis of SNHL in a clinical setting, as ~ 80% of genetic hearing loss is non-syndromic in nature and without the presence of other clinical symptoms or clues to help identify candidate causative gene(s)^3^. Additionally, the high number of deafness-related genes [> 123 genes associated with non-syndromic hearing loss (https://hereditaryhearingloss.org/)] and heterogeneous variants according to ethnic groups has impeded widespread implementation of genetic testing for hearing loss.

Buoyed by the advances in high-throughput genetic sequencing techniques, such as next-generation sequencing (NGS), genetic diagnosis of patients with SNHL has been tremendously expedited. Indeed, exome sequencing (ES) has been increasingly applied to various genetic disorders^6–10^. Overall, the diagnostic yields of ES are estimated at between ~ 25 and ~ 30% among various diseases^6–8,10^. Moreover, the diagnostic yields of ES in monogenic disorders, such as SNHL, reportedly range from ~ 50 to ~ 60%; these values are higher than those in other disorders^6,9^. Further, stepwise and cost-effective genetic analysis protocols employing NGS as the final step of the diagnostic process have been generated for the genetic diagnosis of SNHL^11^. Nevertheless, a considerable number of SNHL subjects still have not benefited from molecular genetic testing in clinics primarily due to inefficiencies associated with sequencing data processing and interpretation.

The time and labor required to evaluate ES data by bioinformaticians cannot maintain pace with the explosive growth in the levels of accumulated sequencing data. Additionally, manual variant prioritization by bioinformaticians can result in variant misdiagnosis or misclassification. Therefore, there is a need for an automated platform capable of annotating and prioritizing candidate variants. Increasing numbers of platforms have been introduced to predict the deleterious effects of variants^12^ and to expedite the evaluation of ES data, including VarFish^13^, exome Disease Variant Analysis (eDiVA)^14^, and Translational Genomics expert (TGex)^15^. Additionally, studies have been conducted on automated genetic diagnosis according to phenotype^16,17^. For example, the Deep PhenomeNET Variant Predictor (DeepPVP)^17^, PhenoPro^18^, Phenoxome^19^, and Phen2Gene^20^ were used to predict causative variants based on phenotype. Benchmark data were developed to validate the performance of these automatic variant prioritization tools using a synthetic patient population^17^ or clinical cohorts with heterogeneous phenotypic entities^19,20^. However, the diagnostic performance of automated and phenotype-driven variant prioritization tools has not been compared with that of human bioinformaticians. In addition, due to the heterogeneous disease entities of previous cohorts, it has not been possible to estimate the diagnostic yield for a single phenotypic disease that could be compared with previous published data.

SNHL, which exhibits a mostly monogenic Mendelian etiology with extreme etiologic heterogeneity, represents an ideal model disorder for assessing an automated prioritization system for identifying causative variants from ES data. We hypothesized that interpretation of ES data from SNHL patients could be expedited by an automated, phenotype-driven, variant prioritization system (EVIDENCE). To test this hypothesis, we used EVIDENCE to analyze ES data from 263 SNHL subjects, with the primary outcome being comparison of the accuracy of variant prioritizations generated by EVIDENCE with the accuracy of prioritizations generated by human bioinformaticians. The secondary outcome was the concordant call rates according to the pathogenic criteria of variants based on the 2015 American College of Medical Genetics and Genomics-Association for Molecular Pathology (ACMG-AMP) guidelines^21^. Additionally, we applied EVIDENCE to evaluate particularly challenging cases reportedly carrying only variants of uncertain significance (VUSs) according to human bioinformaticians. We report a distinct attempt at interpreting ES data from patients with SNHL using automated prioritization of candidate variants.

## Materials and methods

### Participants

This study was approved by the Institutional Ethics Committee of Seoul National University Bundang Hospital (SNUBH; IRB-B-1007-105-402) and the Seoul National University Hospital (SNUH; IRBY-H-0905-041-281). Written informed consent was obtained from patients or their legal representatives in the case of minors. All study protocols complied with the regulations of the Institutional Ethics Committee of Seoul National University Hospital.

**Figure 1:**
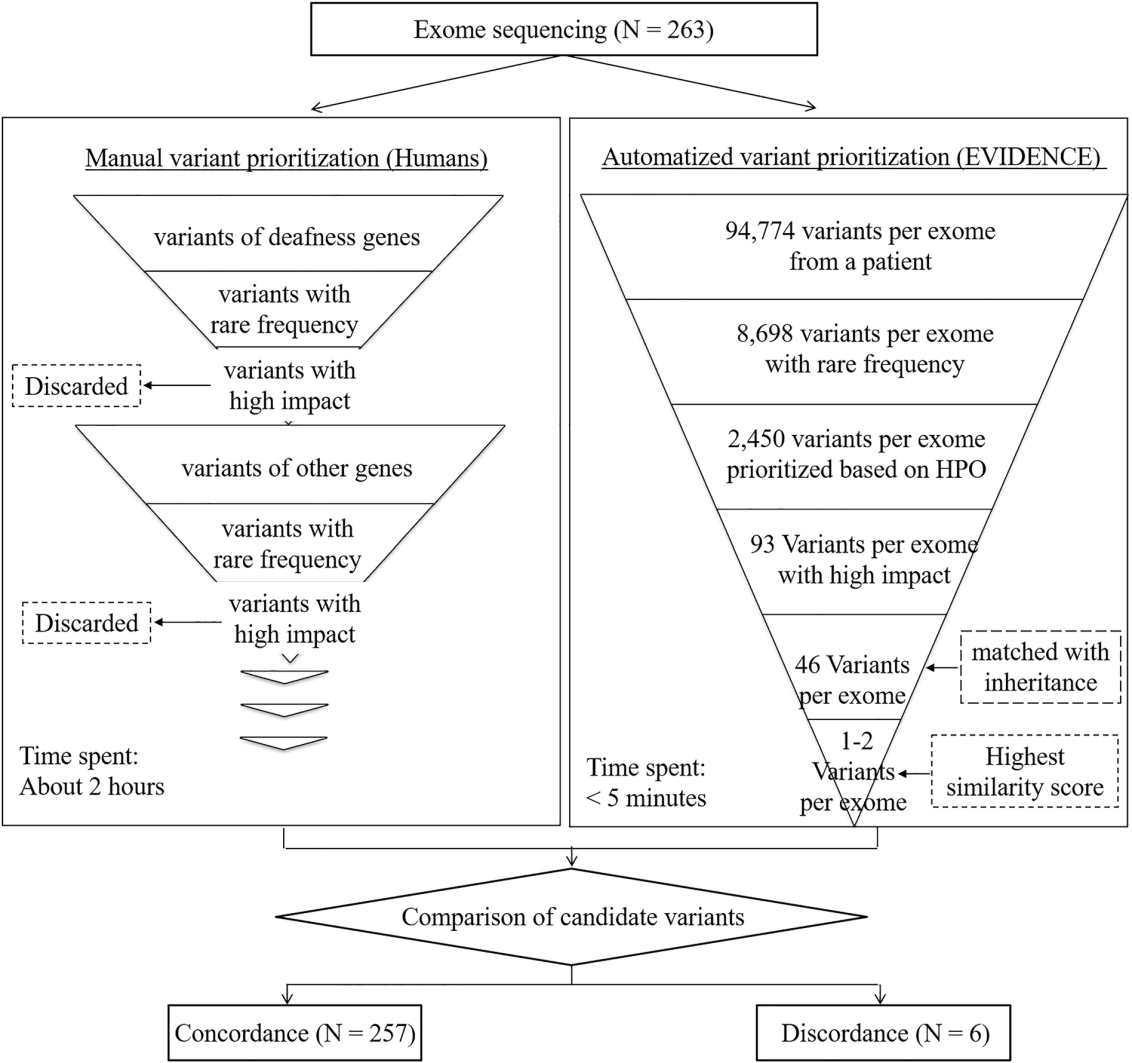
Human and EVIDENCE variant prioritization. A total of 263 unrelated probands from the SNUH and SNUBH sensorineural hearing loss cohort were evaluated using exome sequencing (ES). The ES data was analyzed by human bioinformaticians and using an automated variant prioritization system (EVIDENCE). The prioritization of the variants was compared. The concordant call rate of either prioritized variants or the absence of candidate variants among the entire cohort between humans and EVIDENCE was 97.72% (257/263).

Patients with mild or more severe degrees of SNHL were enrolled. Pure-tone audiometry was performed, and patients with conductive hearing loss were excluded. Tympanic endoscopic examination was conducted, and only the patients with normal tympanic membranes were included. The inheritance pattern was determined based on the segregation study with Sanger sequencing. Sporadic cases were considered as autosomal recessive (AR) if the variants were known to have AR inheritance. A total of 263 unrelated probands from our SNUH and SNUBH SNHL cohort were evaluated using ES, as previously described (Fig. 1)^22^. Sanger sequencing confirmed the presence of all variants listed in Supp. Table S1.

### Variant filtering and prioritization

#### Automated variant prioritization using EVIDENCE

EVIDENCE (https://3billion.io/) is a software package developed to prioritize and interpret variants based on patient phenotype and perform variant classification^23^. This system involves three major steps: variant filtration, classification, and similarity scoring according to patient phenotype (Fig. 1).

First, we used gnomAD v3.1. 1 (http://gnomad.broadinstitute.org/) as a population genome database and the 3billion genome database (https://3billion.io/) to estimate allele frequency. Common variants with minor allele frequencies of > 5% in any subpopulation except for founder populations, such as Finnish and Jewish, were filtered out in accordance with BA1 criterion of the ACMG guidelines^21^. In addition, the exceptional cases reported as BA1 or BS1 variants were also excluded^24^.

Second, we extracted evidence of data on the pathogenicity of variants, including gene function, domain of interest, disease mechanism, inheritance pattern, and clinical relevance, from the scientific literature and disease databases, including OMIM (Access date: August 2020, www.omim.org), ClinVar (Access date: August 2020, https://www.ncbi.nlm.nih.gov/clinvar/), and UniProt (Access date: August 2020, https://www.uniprot.org/). Evaluation of predicted functional or splicing effects and the degree of evolutionary conservation of the identified variants was performed with several in silico tools, including REVEL, ada_score using AdaBoost, and rf score, using the random forest algorithm^25,26^. The reference articles on the variant information including de novo occurrence, functional studies, and segregation data were daily reviewed by clinical geneticists affiliated with 3 billion and updated in EVIDENCE accordingly. Scores > 0.5 in each tool predicted detrimental effects on the variant. Variant pathogenicity was classified and prioritized according to ACMG guidelines^21^. EVIDENCE was used to prioritize variants classified as pathogenic, likely pathogenic, or VUS according to ACMG guidelines, with these variants categorized into three tiers according to their Bayesian score^27^. The first tier includes variants scoring > 0.9, the second > 0.499, and the third > 0.1.

Third, the clinical phenotype(s) of the proband was translated into a corresponding standardized human phenotype ontology (HPO) term and the similarity associated with rare genetic diseases was measured^28,29^. We calculated the similarity score between patient phenotype and symptoms associated with disease caused by prioritized variants according to ACMG guidelines. The processes associated with genetic diagnosis, including processing of raw genomic data, variant prioritization, and phenotype-to-disease similarity measurements, were integrated and automated into a computational framework. The variants were ranked higher according to their increased similarity score based on associations with patient phenotype and disease within each tier. Variants with the highest similarity score within the highest tier were ultimately selected.

#### In silico synthetic cases

To access the EVIDENCE diagnostic yield, we generated 21 synthetic exomes. About 60,000–90,000 common variations, with a minor allele frequency (MAF) > 10% in any subpopulation, were sampled from the GRCh27 phase-3 exomes from the 1000 genome project. Twenty-one of the GRCh27 phase-3 exome VCF files were synthesized using these common variants. Deafness variants were inserted into each synthesized exome VCF file. The deafness variants were selected from previously identified pilot variants, which were classified as pathogenic or likely pathogenic variants in ClinVar (Supp. Table S2)^24^. The variants were prioritized for the 21 synthetic cases using EVIDENCE and Exomiser^30^.

#### Manual prioritization by humans

Twelve persons who expertise in genetic hearing loss and variant prioritization in ES data (S.Y. K., S.L., G.H.S., B.J.K., D.Y.O., J.H.H., M.K.P., S.L., B.K., N.Y., N.J.K., and B.Y.C.) were independently reviewed the prioritized variants and discussed to determine the final candidate variants. The variant prioritization process used in this study was previously described^11^. First, the deafness genes listed in the intra-laboratory database were evaluated for the presence of causative variants. If no causative variants were identified, ES data of other genes were analyzed for the presence of rare variants with deleterious effects. Variants were prioritized based on 2015 ACMG–AMP guidelines for the interpretation of sequence variants^21^. For wider implications of our results and to keep pace with other Mendelian disorders where disease-specific variant interpretation guidelines were not provided, we did not employ the expert specification of the ACMG/AMP variant interpretation guidelines specifically for genetic hearing loss in the final variant classifications^24^. Briefly, the MAF of the variants was accessed using 1000 Genomes (Access date: August 2020, https://www.ncbi.nlm.nih.gov/variation/tools/1000genome), GO-ESP (Access date: August 2020, http://evs.gs.washington.edu/EVS/), GnomAD v3.1.1 (http://gnomad.broadinstitute.org/), and Korean Reference Genome Database [KRGDB; comprising 1722 Korean individuals (3444 alleles) (Access date: August 2020, http://coda.nih.go.kr/coda/KRGDB/index.jsp)]. Initially, variants of any subpopulation with an MAF > 0.05, except for populations with founder alleles, were excluded. Pathogenic variants were inspected according to the literature, ClinVar (Access date: August 2020), or the Deafness Variation Database (Access date: August 2020, http://deafnessvariationdatabase.org/). Then, variants in the total population with an MAF > 0.005 for AR and ≥ 0.001 for autosomal dominant (AD) were further excluded, in accordance with the BA1 criteria of the expert specification of the ACMG/AMP variant interpretation guidelines specifically for genetic hearing loss^24^. SIFT (Access date: August 2020, http://sift.jcvi.org/), PolyPhen2 (Access date: August 2020, http://genetics.bwh.harvard.edu/pph2/), and/or MutationTaster (Access date: August 2020, http://www.mutationtaster.org/) were used for in silico prediction of damage to the function of the resultant protein.

### Comparison of variant prioritization results generated by humans and by EVIDENCE

Variants prioritized by human bioinformaticians and EVIDENCE were compared, and concordant cases were defined as those with identically prioritized variants between humans and EVIDENCE. For cases with multiple VUSs, only cases where all of the variants prioritized by humans and EVIDENCE matched were classified as concordant cases. Cases involving unmatched variants among the lists obtained from the two methodologies were designated as discordant cases. The concordant call rate was calculated according to the variant classification based on ACMG guidelines^21^. For the discordant cases, the variants prioritized by both humans and EVIDENCE were re-evaluated by bioinformaticians.

### Multiplex ligation-dependent probe amplification (MLPA) of stereocilin (STRC)

The mild-to-moderately hearing-impaired probands with only VUS or no possible pathogenic variant were further subjected to MLPA to detect copy number variations (CNVs) encompassing *STRC*^31^. Single heterozygous *STRC* variants were confirmed using long-range nested polymerase chain reaction (PCR) in order to avoid contamination by a pseudogene^31^.

## Results

### Variant prioritization by humans

We found that 50.19% (132/263) of SNHL probands carried candidate variants, with no candidate variants identified in the remaining 49.81% (131/263) from ES data analyzed by humans (Table 1). None of the 131 SNHL probands manifested other syndromic features except for SNHL, while a total of 190 prioritized variants were detected from the 132 SNHL probands of 121 nonsyndromic SNHL and 11 syndromic SNHL, and 50 (50/190, 26.31%) were classified as pathogenic, 69 (69/ 190, 36.32%) as likely pathogenic, and 71 (71/190, 37.37%) as VUS, according to the 2015 ACMG guidelines (Table 2).

**Table 1:** Final variant interpretation results of cohort probands (n [%]).

	Final	Human	EVIDENCE	Concordant probands	Discordant probands
Number of probands with prioritized variants	136 (51.71)	132 (50.19)	134 (50.95)	130 (95.59)	2 by human, 4 by EVIDENCE
**Inheritance**					
Autosomal recessive	83	83	81	81	2
Autosomal dominant	51	47	51	47	4
mitochondrial	1	1	1	1	0
X-linked	1	1	1	1	0
Not found	127 (48.29)	131 (49.81)	129 (49.05)	127 (100)	0
Total	263	263	263	257 (97.72)	6 (2.28)

**Table 2:** The ACMG 2015 classifications of prioritized variants (n [%]).

	Human	EVIDENCE	Concordant	Discordant variants	Final candidate variants
Pathogenic	50 (26.31)	55 (28.35)	50 (92.59)	4 (7.41) of EVIDENCE	54 (27.84)
Likely pathogenic	69 (36.32)	67 (34.54)	67 (97.10)	2 (2.90) of humans	69 (35.57)
Variant of uncertain significance	71 (37.37)	72 (37.11)	71 (100.00)	0	71 (36.60)
Total	190	194	188 (96.91)	6 (3.09) (2 of humans and 4 of EVIDENCE)	194

The addition of molecular genetic testing that enabled the identification of pathogenic CNVs revealed variants in an additional 19 probands (19/263, 7.22%) among the 131 undiagnosed probands (Supp. Table S3), leading to a total diagnostic yield of 57.41%. Of these 19 probands, 10 (10/263, 3.8%) carried one copy of a CNV in a *trans* configuration with a single heterozygous point mutation detected by ES. For these 10 patients, completion of molecular genetic diagnosis was only possible after the implementation of MLPA encompassing *STRC*, ultimately leading to the diagnosis of compound heterozygosity and a point mutation in *STRC.* These point mutations in *STRC* were further confirmed by a long-range nested PCR. SNHL in the other nine probands that had been undiagnosed using ES data (9/263, 3.4%) was exclusively identified by CNVs revealed within the DFNB16 locus (*n* = 6), DFNX2 locus (*n* = 2; SB332-653 and SB430-834), and from chr3q13.11 to chr3q13.31 (*n* = 1; SB318-627).

### Variant prioritization by EVIDENCE

All the deafness variants from the 21 in silico cases were correctly prioritized using EVIDENCE (Supp. Table S2). However, the pathogenic variants of 3 of 21 in silico cases were not prioritized in Exomiser. Three in silico cases had variants of *GJB2* c.101T>C and *GJB2* c.109G>A. For clinical patients, EVIDENCE prioritized 190 candidate variants from the 134 SNHL probands (134/ 263, 50.95%) (Tables 1, 2) at least 24-fold faster than humans (< 5 min vs. 2 h, respectively) and provided equivalent diagnostic yield relative to humans (50.19%) (P = 0.931, chi-squared test).

Two AD variants from three SNHL probands (SB316-522, and SB422-823) prioritized by EVIDENCE were subsequently rejected based on phenotype–genotypic correlations (Table 3). Specifically, *gap junction protein β3* (*GJB3*) c.538C>T was prioritized by EVIDENCE for SB316-522; however, SB316-522 showed enlarged vestibular aqueduct (EVA; unilateral) with Mondini deformity (bilateral), which could not be explained by *GJB3* variants. Similarly, *protein tyrosine phosphatase non-receptor type 1* (*PTPN11*) c.1001T>A was prioritized by EVIDENCE, but this was incompatible to the phenotype of auditory neuropathy spectrum disorder (ANSD) in SB422-823. EVIDENCE selected *GJB3* c.538C>T for SB316-522, because this variant met PVS1, PM2, and PP5 criteria based on multiple lines of data and was thus classified as a pathogenic variant according to the 2015 ACMG-AMP guidelines.

**Table 3:** The sensorineural hearing loss probands whose candidate variants were detected by humans.

Patient ID	Gender/age	Clinical phenotype	Data analyst	Gene	HGVS nomenclature		Zygosity	ACMG/AMP 2015	
					Nucleotide change	protein change		Classification	Criteria
SB316–522	F/9m	Unilateral EVA both Mondini deformity	Hu	*SLC26A4*	NM_000441.1:c.2168A>G	NP_000432:p.His723Arg	Heterozygote	Likely pathogenic	PM1, PM2, PM3, PP2, PP3, PP5
			AI	*GJB3*	NM_024009.2:c.538C>T	NP_076872.1:p.Arg180Ter	Heterozygote	Pathogenic	PVS1, PM2, PP5
SB422–823	M/18 m	Auditory neuropathy	Hu	*OTOF*	NM_001287489.1:c.2521G>A	NP_919224.1:p.Glu841Lys	Heterozygote	Likely pathogenic	PM2, PM3, PP3, PP4
			AI	*PTPN11*	NM_002834.3:c.1001T>A	NP_002825 :p.Leu334Gln	Heterozygote	VUS	PM2, PP2, PP3

*EVA* enlarged vestibular aqueduct, *Hu* humans, *AI* EVIDENCE.

### Cooperative prioritization of variants by humans and EVIDENCE

Comprehensive analysis by both humans and EVIDENCE revealed that 51.71% (136/263) of SNHL probands carried one or more candidate causative variants (194 prioritized variants), of which 54 (54/194, 27.84%) were classified as pathogenic, 69 (69/194, 35.57%) as likely pathogenic, and 71 (71/194, 36.60%) as VUS, according to the 2015 ACMG guidelines (Tables 1, 2, Supp. Table S2). The concordant call rate of either prioritized variants or the absence of candidate variants among the entire cohort between humans and EVIDENCE was 97.72% (257/263) (Table 1). According to the variant classifications, the concordance rate was 92.59% (50/54) for pathogenic variants, 97.10% (67/69) for likely pathogenic variants, and 100.00% (71/71) for VUS, with no significant difference observed in the concordance rate based on the variant classification (P = 0.065, chi-squared test). For discordant cases, two causative variants were solely prioritized by humans (Table 3), whereas four pathogenic variants from four SNHL probands were exclusively identified and confirmed by EVIDENCE (Table 4).

**Table 4:** The pathogenic variants detected exclusively by EVIDENCE.

Patient ID	Gender/age	Clinical phenotype	Gene	HGVS nomenclature		Zygosity	ACMG/AMP 2015	
				Nucleotide change	Protein change		**Classification**	**Criteria**
SB308–611	M/7m	Hearing loss, heart mur-mur	*PTPN11*	NM_002834.3:c.922A>G	NP_002825.3:p.Asn308Asp	Heterozygote	Pathogenic	PS2, PM1, PM2, PM5, PP1, PP2, PP3, PP5
SH271–631	M/10m	Hearing loss, pulmonary stenosis	*PTPN11*	NM_002834.3:c.922A>G	NP_002825.3:p.Asn308Asp	Heterozygote	Pathogenic	PS2, PM1, PM2, PM5, PP1, PP2, PP3, PP5
SH250–590	F/0	Profound hearing loss	*PTPN11*	NM_002834.3:c.836A>G	NP_002825.3:p.Tyr279Cys	Heterozygote	Pathogenic	PS2, PM1, PM2, PM5, PM6, PP2, PP3, PP5
SB542–1014	M/8m	Mixed hearing loss, mandibulofacial anomaly	*EFTUD2*	NM_001258353.1:c.271+1G>A	NP_001245282.1:p.Glu91Aspfs*24	Heterozygote	Pathogenic	PSV1, PS2,PS3, PM2, PP4

### Causative variants identified only by humans

Two SNHL probands carried a pathogenic variant of *solute carrier 26A4* (*SLC26A4*) c.2168A>G (SB316-522) and a likely pathogenic variant in *otoferlin* (*OTOF*) c.2521G>A (SB422-823), prioritized only by humans (Table 3). Both c.2168A>C of *SLC26A4* and c.2521G>A of *OTOF* were detected as single heterozygotes. Although these variants did not meet the criteria for AR inheritance, the phenotypes associated with SB316-522 and SB422-823 were EVA (unilateral) with Mondini deformity (bilateral) and prelingual ANSD with the radiologically normal cochlear nerve, respectively, and highly suggestive of causal variants in *SCL26A4* (DFNB4) and *OTOF* (DFNB9) in Koreans. However, EVIDENCE prioritized a variant classified as a pathogenic variant (*GJB3*:c.539C>T) and a variant that complied with the AD inheritance pattern (*PTPN11*:c.1001T>A).

### Pathogenic variants identified only by EVIDENCE

Four pathogenic variants were exclusively identified by EVIDENCE (Table 4). In addition to its speed, EVIDENCE showed efficacy in the molecular diagnosis of rare syndromic deafness. For example, two *PTPN11* variants of c.922A>G and c.836A>G from three probands were identified by EVIDENCE, none of whom (SH 271–631, SH 250–590, and SB308–611) showed abnormal facial features or skeletal malformations associated with Noonan syndrome, but demonstrated only severe SNHL. Other features were not sufficient to phenotypically suspect Noonan syndrome without molecular genetic confirmation. Additionally, SH 271–631 and SB308–611 did not manifest any syndromic features outside of congenital pulmonary artery stenosis. Moreover, SH 250–590 also did not demonstrate any syndromic features outside of multiple dark spots (lentigines) throughout the body. All of the probands underwent cochlear implantation (CI) and demonstrated favorable hearing outcomes. SH 271–631 and SB308–611 underwent CI at 11 months, with a Categories of Auditory Performance (CAP) score of 5 at 1 year post-operation. SH 250–590 underwent CI at 13 months, with a CAP score of 5 at 15 months post-operation. One *EFTUD2* variant of c.271+1G>A was identified by EVIDENCE^32^. A proband (SB542–1014) carrying the *EFTUD2* variant showed mixed hearing loss, mandibulofacial anomaly, and congenital heart defect, and the pathogenicity of c.271+1G>A was validated by a minigene assay^32^. Humans were unable to prioritize any variants related to rare syndromic hearing loss in these four SNHL probands. Thus, four SNHL probands, who were not previously reported to harbor any candidate variant by humans, were identified as carrying a pathogenic variant by EVIDENCE. Therefore, the proportion of the SNHL probands who remained “undiagnosed” after ES by humans was reduced from 49.81% (131/263) to 48.29% (127/263) through the assistance of EVIDENCE.

## Discussion

This study notably validated the application of automated phenotype-driven analysis software using clinical data from the large-scale hearing loss cohort comprising 263 real patients rather than hypothetical subjects. Although the candidate variant prioritization by humans is not a gold standard method, it is a conventional method for diagnosis of genetic hearing loss. To improve the diagnostic accuracy in manual curations, twelve expertized persons in clinical genetics and genetic hearing loss were involved in manual curation process and conducted consensus discussion more than three times. Moreover, in silico analysis were conducted and the results were compared with other program of Exomiser. In addition to the definitively diagnosed cases carrying exclusively pathogenic or likely pathogenic variants, complex cases harboring single or multiple VUS could also be analyzed by EVIDENCE. Given the increasing number of these complex cases, the findings of the present study promote the clinical use of automated phenotype-driven analysis software for diagnosing and genetically testing SNHL patients.

EVIDENCE was able to prioritize candidate variants associated with SNHL with a 97.72% (257/263) concordance rate with variants identified by experienced human bioinformaticians. In terms of molecular diagnostic yield for SNHL using ES data, EVIDENCE narrowly outperformed human bioinformaticians [50.95% (134/263) vs. 50.19% (132/263)]. Notably, EVIDENCE unveiled pathogenic variants in four SNHL probands that would not have been identified by human bioinformaticians. However, human bioinformaticians managed to identify most of the convincing candidate variants from three SNHL probands after referring to predefined, specific genotype–phenotype correlations, which was not possible using EVIDENCE. Moreover, the combined results of humans and EVIDENCE resulted in an ES diagnostic yield of 51.71% (136/263).

We found that EVIDENCE processed variant prioritization from ES data about 24-fold faster than human bioinformaticians (~ 5 min vs. 2 h). Indeed, excessive time would have been required for manual analyses conducted by unskilled bioinformaticians. The time spent curating candidate disease-causing variants in ES data was estimated as ~ 54 min (range 5–223 min) per variant, and ~ 81 h was predicted as the time required for manual prioritization of variant in ES data based on an estimated 90–127 genetic variants curated from each individual^33^. To expedite the analysis of ES data, multiple programs, including Exomiser or Genomiser tools^34,35^ and Phevor^36,37^, have been developed. The diagnostic yield of these automated methods is considered comparable with that of manual analyses, although failure to curate a candidate variant could happen with automated software due to inappropriate thresholds related to phenotypic cut-off filters^37^. Given that the diagnostic yield of ES of hearing loss has been superior to that of other disorders (55% vs. 28.8% for overall disorders)^6^, automated phenotype-driven analysis of ES data could be clinically applicable to patients with hearing loss and presumably with the potential for relatively higher diagnostic yield in other diseases. Although previous studies validated phenotype-driven analysis software in comparison with conventional manual analysis^17,37^, no previous studies analyzed patients with SNHL in this context. The syndromic features of SNHL, including facial dysmorphisms and developmental delay, do not become obvious often until later stages; thus, genetic diagnosis of neonatal SNHL could predate manifestation of the syndromic features, as demonstrated by our four cases exclusively diagnosed by EVIDENCE.

Focusing on the pathogenic or likely pathogenic variants, the concordance rate of EVIDENCE with analysis by human bioinformatician was 95.12% (117/ 123) (Table 2). Notably, EVIDENCE outperformed manual variant prioritization, especially in cases of syndromic deafness. This might be due to the absence of a phenotype or its subclinical syndromic status at the time of genetic diagnosis in these syndromic patients, which is usually no later than the age of 1 year. Thus, it is not infrequent that the clinician could not think of the syndromic SNHL and the variants of causative genes of syndromic SNHL could be discarded. Additionally, the wide spectrum of phenotypes related to syndromic deafness hampers identification of specific candidate causative genes. As a classic example, Noonan syndrome demonstrates various spectrums of clinical features^38,39^. In the present study, three *PTPN11* probands, missed by humans, did not exhibit definite syndromic facial features. Furthermore, genes associated with syndromic hearing loss can be detected, even in patients with non-syndromic hearing loss and with no or subclinical syndromic phenotypes^40^, precluding prediction of a causative gene solely based on a syndromic phenotype. For example, our previous study reported an ANSD patient carrying an *ATP1A3* variant without pathognomonic features and presenting a cerebellar ataxia, areflexia, pes cavus, optic atrophy, and sensorineural hearing loss (CAPOS) phenotype^41^. EVIDENCE could potentially facilitate early diagnosis of such syndromic diseases before patients manifest the definite clinical features. Another proband with an *EFTUD2* splice-site variant was also diagnosed exclusively by EVIDENCE which was retrospectively reviewed by humans and published in another article^32^. Although this proband (SB542-1014) did show syndromic mandibulofacial anomaly and congenital cardiac defect, molecular diagnosis of the *EFTUD2* variant was not made by humans, likely due to the rarity and wide spectrum of the phenotypes of mandibulofacial dysostosis, Guion–Almeida type.

The other two discordant calls between EVIDENCE and humans regarding pathogenic or likely pathogenic variants arose from different interpretations of single heterozygous, AR, likely pathogenic variants, which were exclusively prioritized as causative variants only by humans. Human bioinformaticians can consider these monoallelic recessive alleles as causative variants, relying on the very specific radiological or audiological phenotype. Specifically, unilateral EVA accompanied by both sides of incomplete partition type II (referred to as “Mondini malformations” from SB316-522 and prelingual ANSD from SB422-823) was so distinctive that these phenotypes made the monoallelic variant, detected from their signature gene. We speculate that yet-to-be identified noncoding region variants or CNVs in or encompassing *SLC26A4* and *OTOF* might contribute to these specific phenotypes in a *trans* configuration with the single heterozygous allele. *SLC26A4* c.2168A>G is a well-known recurring pathogenic variant with null function previously demonstrated in an in vitro study^42^. Although *SLC26A4* variants that cause hearing loss have AR inheritance, a number of previous studies demonstrated EVA with monoallelic *SLC26A4* variants^43,44^. These monoallelic *SLC26A4* variants are proposed to cause EVA in combination with either yet-to-be identified pathogenic variants in noncoding regulatory regions of *SLC26A4,* as supported by analysis of EVA-recurrence rates^43–45^, or regulatory genes of *SLC26A4*, such as *EPHA2*^46^. On the other hands, EVIDENCE prioritized *GJB3* c.538C>T as a candidate variant for SB316-522. *GJB3* was first reported as a causative gene for bilateral high-frequency hearing loss^47^, with three additional studies suggesting the pathogenic potential of *GJB3* for hearing loss with uncertain significance^48–50^. However, although *GJB3* c.538C>T co-segregated with hearing loss in two Chinese families as an AD inheritance, one unaffected family member also harbored a monoallelic *GJB3* c.538C>T variant^47^, precluding the confirmation of the pathogenic potential of *GJB3* c.538C>T. Additionally, the MAF in the KRGDB was reported at 0.09% (3/1722 individuals), implying benign pathogenic potential of this variant.

**Figure 2:**
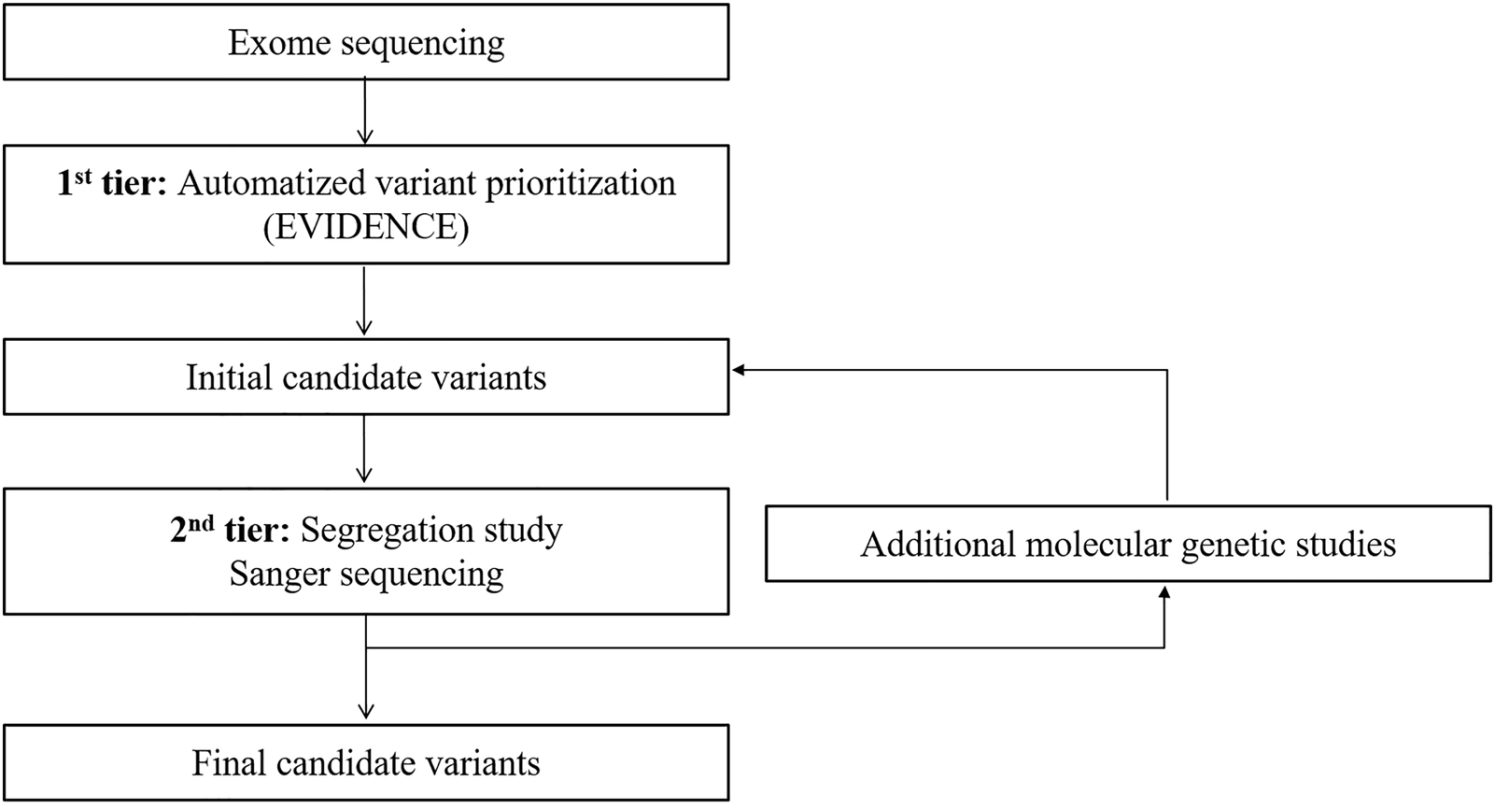
Proposed workflow to reach the molecular diagnosis of genetic hearing loss cases with available exome sequencing (ES) data. The automatized variant prioritization using EVIDENCE is the first-tier analysis, which is followed by the second-tier analyses including segregation study and Sanger sequencing. Additional molecular genetic studies are also required for cases undiagnosed by ES.

Another monoallelic, likely pathogenic variant in the AR gene *OTOF* (c.2521G>A) was prioritized by humans in a proband (SB422-823) with prelingual ANSD. This variant was estimated as the second-most common (as high as 13.6%) *OTOF* variant in *OTOF*-related ANSD (DFNB9) in Koreans^51^. The pathogenicity of single heterozygous *OTOF* variants has been reported in clinical studies^52,53^. Given the etiologic homogeneity of prelingual ANSD, the single heterozygous *OTOF* variant likely contributes to prelingual ANSD in combination with yet-to-be identified variants in the noncoding region of *OTOF* or CNVs encompassing *OTOF*^54^. In the present study, EVIDENCE could not interpret these monoallelic variants in the absence of detailed genotype–phenotype information and data showing the possible presence of variants in a *trans* configuration. Therefore, the second-tier analyses following this variant prioritization by EVIDENCE such as a segregation study (Fig. 2) are mandatory. Additionally, in this study, 19 probands required further molecular genetic studies beyond ES, such as chromosomal and CNV analyses (Fig. 2).. To identify pathogenic genetic deletions, understanding the clinical phenotype of these 19 probands was crucial. Although hearing loss could be a single phenotype in HPO terms, types and degrees of hearing loss can be diverse according to the causal genes. Mild-to-moderate SNHL without any detectable causal variants in known deafness genes could be caused by CNVs in *STRC*^31^. Given this knowledge, 16 probands of DFNB16 were identified as carrying *STRC* large deletions using MLPA. Although ES alone did not enable us to reach a conclusive genetic diagnosis, the *STRC* single heterozygote variant could be a clue for further molecular genetic studies to evaluate the presence of CNVs, in addition to providing information concerning the exclusion of the causal variants in known deafness genes. Indeed, in our cohort, 62.5% (10/16) of DFNB16 probands harbored a single heterozygote *STRC* variant, which was detected in ES. Two probands with genomic deletion in the *POU3F4* upstream region could not be detected in ES. Although no causal variant was selected in ES, the cochlear anomaly of incomplete partition type III in two probands (SB332–653 and SB430–834) provided clues for the diagnosis of DFNX2^55^.

## Conclusion

EVIDENCE facilitated the exploration of candidate variants from ES, and its application saved significant time and effort during variant prioritization and improved the detection rate for pathogenic and likely pathogenic variants of hearing loss. Although it was overall estimated that EVIDENCE expedited the variant prioritization process about 24 fold faster than humans, the exact time required for manual variant prioritization by humans varied significantly for each ES, precluding simply displaying the difference in time and efficiency of prioritization between humans and EVIDENCE in a single number. In addition, due to the relatively high detection rate of hearing loss candidate variants in ES, compared to other disorders, the present EVIDENCE diagnostic yield could not be applied to other genetic disorders. However, this is the largest cohort study that validated the diagnostic yield of a phenotype-driven ES analysis software. Moreover, we performed additional downstream genetic studies beyond ES for patients in whom CNV was suspected, allowing subsequent causative genetic diagnoses. Furthermore, cases with discordant calls between EVIDENCE and humans spotlighted the strength of automated prioritization of candidate variants and also provided guidance as to which direction EVIDENCE should evolve and how manual prioritization should improve. The cooperation of EVIDENCE with clinical geneticists could yield higher diagnostic accuracy and efficiency in analyzing and filtering ES data.

## Supplementary Information

Supplementary Information 1.

Supplementary Information 2.

Supplementary Information 3.

## Data Availability

The raw data of experiments used to support the findings of this study are available from the corresponding author upon request. The variant prioritization using EVIDENCE (https://3billion.io/) is available after registration with cost.

## References

[1] Korver AM (2017). Congenital hearing loss. Nat. Rev. Dis. Primers.

[2] Smith RJ, Bale JF, White KR (2005). Sensorineural hearing loss in children. Lancet.

[3] Morton CC, Nance WE (2006). Newborn hearing screening—a silent revolution. N. Engl. J. Med..

[4] Park JH (2017). Outcome of cochlear implantation in prelingually deafened children according to molecular genetic etiology. Ear Hear..

[5] Song MH (2019). Genetic inheritance of late-onset, down-sloping hearing loss and its implications for auditory rehabilitation. Ear Hear..

[6] Retterer K (2016). Clinical application of whole-exome sequencing across clinical indications. Genet. Med..

[7] Yang Y (2014). Molecular findings among patients referred for clinical whole-exome sequencing. JAMA.

[8] Lee H (2014). Clinical exome sequencing for genetic identification of rare Mendelian disorders. JAMA.

[9] Stark Z (2016). A prospective evaluation of whole-exome sequencing as a first-tier molecular test in infants with suspected monogenic disorders. Genet. Med..

[10] Yang Y (2013). Clinical whole-exome sequencing for the diagnosis of Mendelian disorders. N. Engl. J. Med..

[11] Han JJ (2019). Elucidation of the unique mutation spectrum of severe hearing loss in a Vietnamese pediatric population. Sci. Rep..

[12] Chennen K (2020). MISTIC: A prediction tool to reveal disease-relevant deleterious missense variants. PLoS One.

[13] Holtgrewe M (2020). VarFish: Comprehensive DNA variant analysis for diagnostics and research. Nucleic Acids Res..

[14] Bosio M (2019). eDiVA-Classification and prioritization of pathogenic variants for clinical diagnostics. Hum. Mutat..

[15] Dahary D (2019). Genome analysis and knowledge-driven variant interpretation with TGex. BMC Med. Genom..

[16] Gurovich Y (2019). Identifying facial phenotypes of genetic disorders using deep learning. Nat. Med..

[17] Boudellioua I, Kulmanov M, Schofield PN, Gkoutos GV, Hoehndorf R (2019). DeepPVP: Phenotype-based prioritization of causative variants using deep learning. BMC Bioinform..

[18] Li Z (2019). PhenoPro: A novel toolkit for assisting in the diagnosis of Mendelian disease. Bioinformatics.

[19] Wu C (2019). Rapid and accurate interpretation of clinical exomes using Phenoxome: A computational phenotype-driven approach. Eur. J. Hum. Genet..

[20] Zhao M (2020). Phen2Gene: Rapid phenotype-driven gene prioritization for rare diseases. NAR Genom. Bioinform..

[21] Richards S (2015). Standards and guidelines for the interpretation of sequence variants: A joint consensus recommendation of the American College of Medical Genetics and Genomics and the Association for Molecular Pathology. Genet. Med..

[22] Kim NK (2015). Whole-exome sequencing reveals diverse modes of inheritance in sporadic mild to moderate sensorineural hearing loss in a pediatric population. Genet. Med..

[23] Seo GH (2020). Diagnostic yield and clinical utility of whole exome sequencing using an automated variant prioritization system, EVIDENCE. Clin Genet..

[24] Oza AM (2018). Expert specification of the ACMG/AMP variant interpretation guidelines for genetic hearing loss. Hum. Mutat..

[25] Ioannidis NM (2016). REVEL: An ensemble method for predicting the pathogenicity of rare missense variants. Am. J. Hum. Genet..

[26] Jian X, Boerwinkle E, Liu X (2014). In silico prediction of splice-altering single nucleotide variants in the human genome. Nucleic Acids Res..

[27] Tavtigian SV (2018). Modeling the ACMG/AMP variant classification guidelines as a Bayesian classification framework. Genet. Med..

[28] Greene D, BioResource N, Richardson S, Turro E (2016). Phenotype similarity regression for identifying the genetic determinants of rare diseases. Am. J. Hum. Genet..

[29] Kohler S (2009). Clinical diagnostics in human genetics with semantic similarity searches in ontologies. Am. J. Hum. Genet..

[30] Smedley D (2015). Next-generation diagnostics and disease-gene discovery with the Exomiser. Nat. Protoc..

[31] Kim BJ (2020). Significant Mendelian genetic contribution to pediatric mild-to-moderate hearing loss and its comprehensive diagnostic approach. Genet. Med..

[32] Kim SY, Lee DH, Han JH, Choi BY (2020). Novel splice site pathogenic variant of EFTUD2 is associated with mandibulofacial dysostosis with microcephaly and extracranial symptoms in Korea. Diagnostics (Basel).

[33] Dewey FE (2014). Clinical interpretation and implications of whole-genome sequencing. JAMA.

[34] Smedley D, Robinson PN (2015). Phenotype-driven strategies for exome prioritization of human Mendelian disease genes. Genome Med..

[35] Smedley D (2016). A whole-genome analysis framework for effective identification of pathogenic regulatory variants in Mendelian disease. Am. J. Hum. Genet..

[36] Singleton MV (2014). Phevor combines multiple biomedical ontologies for accurate identification of disease-causing alleles in single individuals and small nuclear families. Am. J. Hum. Genet..

[37] Thuriot F (2018). Clinical validity of phenotype-driven analysis software PhenoVar as a diagnostic aid for clinical geneticists in the interpretation of whole-exome sequencing data. Genet. Med..

[38] Li X (2019). Molecular and phenotypic spectrum of Noonan syndrome in Chinese patients. Clin. Genet..

[39] Tartaglia M (2002). PTPN11 mutations in Noonan syndrome: Molecular spectrum, genotype-phenotype correlation, and phenotypic heterogeneity. Am. J. Hum. Genet..

[40] Bademci G (2016). Variations in multiple syndromic deafness genes mimic non-syndromic hearing loss. Sci. Rep..

[41] Wang J (2017). RNA-sequencing analysis reveals the hepatotoxic mechanism of perfluoroalkyl alternatives, HFPO2 and HFPO4, following exposure in mice. J. Appl. Toxicol..

[42] Jang JH (2014). Identification of novel functional null allele of SLC26A4 associated with enlarged vestibular aqueduct and its possible implication. Audiol. Neurootol..

[43] Mey K (2019). Association of SLC26A4 mutations, morphology, and hearing in pendred syndrome and NSEVA. Laryngoscope.

[44] Yang T (2007). Transcriptional control of SLC26A4 is involved in Pendred syndrome and nonsyndromic enlargement of vestibular aqueduct (DFNB4). Am. J. Hum. Genet..

[45] Choi BY (2009). Segregation of enlarged vestibular aqueducts in families with non-diagnostic SLC26A4 genotypes. J. Med. Genet..

[46] Li M (2020). Digenic inheritance of mutations in EPHA2 and SLC26A4 in Pendred syndrome. Nat. Commun..

[47] Xia JH (1998). Mutations in the gene encoding gap junction protein beta-3 associated with autosomal dominant hearing impairment. Nat. Genet..

[48] He LQ (2005). Intracellular distribution, assembly and effect of disease-associated connexin 31 mutants in HeLa cells. Acta Biochim. Biophys. Sin. (Shanghai).

[49] Xia K (2010). Trafficking abnormality and ER stress underlie functional deficiency of hearing impairment-associated connexin-31 mutants. Protein Cell.

[50] Yao G (2013). Novel mutations of SLC26A4 in Chinese patients with nonsyndromic hearing loss. Acta Otolaryngol..

[51] Kim BJ (2018). Mutational and phenotypic spectrum of OTOF-related auditory neuropathy in Koreans: Eliciting reciprocal interaction between bench and clinics. J. Transl. Med..

[52] Wang J (2011). Variants of OTOF and PJVK genes in Chinese patients with auditory neuropathy spectrum disorder. PLoS One.

[53] Varga R (2003). Non-syndromic recessive auditory neuropathy is the result of mutations in the otoferlin (OTOF) gene. J. Med. Genet..

[54] Chang MY (2015). Refinement of molecular diagnostic protocol of auditory neuropathy spectrum disorder: Disclosure of significant level of etiologic homogeneity in Koreans and its clinical implications. Medicine (Baltimore).

[55] Choi JW (2015). De novo large genomic deletions involving POU3F4 in incomplete partition type III inner ear anomaly in East Asian populations and implications for genetic counseling. Otol. Neurotol..

