# Supplementary material for: Powerful use of automated prioritization of candidate variants in genetic hearing loss with extreme etiologic heterogeneity
Source: Sci Rep. 2021 Sep 30;11:19476. doi: 10.1038/s41598-021-99007-3 (PMC8484668; doi:10.1038/s41598-021-99007-3)
Supplement: Supplementary file 3 — Supplementary Information 3. [file 41598_2021_99007_MOESM3_ESM.docx]

**Table S3.** The sensorineural hearing loss probands diagnosed by additional molecular genetic studies

| Patient ID | Gender/Age | Gene (ES) | HGVS Nomenclature | | ACMG/AMP 2015 | | Additional Tests | Gene | Zygosity |
| --- | --- | --- | --- | --- | --- | --- | --- | --- | --- |
|  |  |  | **Nucleotide Change** | **Protein Change** | **Classification** | **Criteria** |  |  |  |
| SB291-584 | M/20 | (Not found) |  |  |  |  | MLPA | *STRC* | Homozygous large deletion |
| SB262-516 | F/5 | *(Not found)* |  |  |  |  | MLPA | *STRC* | Homozygous large deletion |
| SB267-524 | M/23 | *(Not found)* |  |  |  |  | MLPA | *STRC* | Homozygous large deletion |
| SB286-570 | F/18 | *STRC* | NM_153700.2:c.4226_4229delTGGG/insCA | NP_714544:p.Leu1409Serfs*25 | Pathogenic | PVS1, PM2, PP1 | MLPA | *STRC* | Heterozygous large deletion |
| SB248-490 | F/9 | *STRC* | NM_153700.2:c.5141_5142delTG | NP_714544:p.Val1714Glyfs*5 | Likely pathogenic | PVS1, PM2 | MLPA | *STRC* | Heterozygous large deletion |
| SB287-572 | F/12 | *STRC* | NM_153700.2:c.4816_4817insC | NP_714544:p.Leu1606Profs*25 | Likely pathogenic | PVS1, PM2 | MLPA | *STRC* | Heterozygous large deletion |
| SB323-637 | F/1 | *STRC* | NM_153700.2:c.4226_4229delTGGG/insCA | NP_714544:p.Leu1409Serfs*25 | Likely pathogenic | PVS1, PM2, PP1 | MLPA | *STRC* | Heterozygous large deletion |
| SH110-228 | M/7 | *STRC* | NM_153700.2:c.4057C>T | NP_714544:p.Gln1353* | Pathogenic | PVS1, PM2, PP5 | MLPA | *STRC* | Heterozygous large deletion |
| SH157-341 | M/27 | *(Not found)* |  |  |  |  | MLPA | *STRC* | Homozygous large deletion |
| SH177-394 | F/2 | *(Not found)* |  |  |  |  | MLPA | *STRC* | Homozygous large deletion |
| SB318-627 | M/5m | *(Not found)* |  |  |  |  | Chromosomal analysis | *Chr deletion* | |
| SB355-693 | F/12m | *STRC* | NM_153700.2:c.4226_4229delTGGG/insCA | NP_714544:p.Leu1409Serfs25 | Pathogenic | PVS1, PM2, PP1 | MLPA | *STRC* | Heterozygous large deletion |
| SB332-653 | M/5m | *(Not found)* |  |  |  |  | Quantitative PCR | *POU3F4* | hemizygous large deletion |
| SB135-228 | F/13 | *STRC* | NM_153700.2:c.583C>T | NP_714544:p.Gln195* | Likely pathogenic | PVS1, PM2, PM3, PP1 | MLPA | *STRC* | Heterozygous large deletion |
| SB118-210 | M/5 | *STRC* | NM_153700.2:c.4552G>A | NP_714544:p.Gly1518Ser | VUS | PM2, PP3 | MLPA | *STRC* | Heterozygous large deletion |
| SB269-530 | M/7 | *STRC* | NM_153700.2:c.4057C>T | NP_714544:p.Gln1353* | Pathogenic | PVS1, PM2, PP5 | MLPA | *STRC* | Heterozygous large deletion |
| SB430-834 | M/11m | *(Not found)* |  |  |  |  | Quantitative PCR | *POU3F4* | hemizygous large deletion |
| SB410-803 | F/28 | *(Not found)* |  |  |  |  | MLPA | *STRC* | Homozygous large deletion |
| SB433-840 | M/19 | *STRC* | NM_153700.2:c.2614C>T | NP_714544:p.Pro872Ser | VUS | PM2 | MLPA | *STRC* | Heterozygous large deletion |
